# Reading activity prevents long-term decline in cognitive function in older people: evidence from a 14-year longitudinal study

**DOI:** 10.1017/S1041610220000812

**Published:** 2021-01

**Authors:** Yu-Hung Chang, I-Chien Wu, Chao A. Hsiung

**Affiliations:** 1 Department of Public Health, China Medical University, Taichung, Taiwan; 2 Institute of Population Health Sciences, National Health Research Institutes, Zhunan, Taiwan

**Keywords:** cognitive function, reading, education

## Abstract

**Objective:**

This study examined the effect of daily life reading activity on the risk of cognitive decline and whether the effect differs regarding education levels.

**Design:**

A longitudinal study with 6-, 10-, and 14-year follow-up.

**Setting:**

Face-to-face interviews with structured questionnaires at home.

**Participants:**

A representative sample of 1,962 Taiwanese community-dwelling older persons aged 64 and above, followed up in four waves of surveys over 14 years.

**Measurements:**

Baseline reading frequencies were measured based on a scale of leisure activity. The Short Portable Mental Status Questionnaire was used to measure cognitive performance. We performed logistic regression to assess associations between baseline reading and later cognitive decline. Interaction terms between reading and education were to compare the reading effects on cognitive decline at different education levels.

**Results:**

After adjusting for covariates, those with higher reading frequencies (≥1 time a week) were less likely to have cognitive decline at 6-year (adjusted odds ratio [AOR]: 0.54; 95% confidence interval [CI]: 0.34–0.86), 10-year (AOR: 0.58, 95% CI: 0.37–0.92), and 14-year (AOR: 0.54, 95% CI: 0.34–0.86); in a 14-year follow-up, a reduced risk of cognitive decline was observed among older people with higher reading frequencies versus lower ones at all educational levels.

**Conclusions:**

Reading was protective of cognitive function in later life. Frequent reading activities were associated with a reduced risk of cognitive decline for older adults at all levels of education in the long term.

## Introduction

High level of cognitive functional capacity is one of the major components of successful aging (Rowe and Kahn, 1997). The World Health Organization (WHO) highlighted the importance of cognitive function in determining health in its policy framework on active aging (WHO, 2002). Cognitive decline is a normal process of aging (Harada *et al.*, 2013). Mild cognitive impairment is a greater cognitive decline beyond one’s age without interfering with daily life activities; dementia is characterized by more severe and widespread cognitive impairments that substantially influence daily functions (Gauthier *et al.*, 2006). The prevalence of mild cognitive impairment over the age of 60 ranges from 6.7% to 25.2% and increases with age (Petersen *et al.*, 2018). The number of persons with dementia worldwide was estimated to be 46.8 million in 2015, resulting in 818 billion USD worth of societal costs (Prince, 2015). In East Asia, the region of the most populations living with dementia, the prevalence of dementia in older people aged 60 or older was 4.5% in 2015 (Prince, 2015). In Taiwan, the prevalence of cognitive impairment among older adults aged 65 and over was 22% (Wu *et al.*, 2011) and that of dementia was 5.7% (Wu *et al.*, 2013), respectively. Dementia affected 210,000 Taiwanese older adults by 2020 and is projected to affect 710,000 by 2060 (Wu *et al.*, 2013).

Cognitive impairments and dementia could predict premature death (Sachs *et al.*, 2011; Todd *et al.*, 2013). Loss of cognitive function was associated with reduced functional abilities of daily living, compromised well-being, and lower life satisfaction (Comijs *et al.*, 2005; Mehta *et al.*, 2002; St. John and Montgomery, 2010). In addition to normal aging, cognitive decline and impairments may be induced by lack of practice (Mackinnon *et al.*, 2003), anxiety, depression or other neuropsychiatric symptoms (Lara *et al.*, 2017; Palmer *et al.*, 2011; Potvin *et al.*, 2011), vascular factors (Baumgart *et al.*, 2015; Debette *et al.*, 2011), substance use and medication (Anstey *et al.*, 2009; Fox *et al.*, 2011), loneliness (Boss *et al.*, 2015), low social support, and isolation (Kelly *et al.*, 2017). The process of cognitive decline could be slowed by involvement in regular physical activity (Ma *et al.*, 2017; Northey *et al.*, 2018), avoiding smoking (Baumgart *et al.*, 2015), adopting particular dietary patterns and nutrient intake (Lee *et al.*, 2017; Solfrizzi *et al.*, 2017), or more engagement in social life and leisure activity (Ghisletta *et al.*, 2006; Glei *et al.*, 2005).

Cognitively stimulating activities, or intellectual activities, are recognized as a lifestyle factor in preserving cognitive function in the aged (Hultsch *et al.*, 1999; Mackinnon *et al.*, 2003). Studies have shown that cognitively stimulating or intellectual activities, including reading, watching TV, listening to radio, playing games, puzzling, or gambling was associated with a reduced risk of cognitive decline in later life (Gallucci *et al.*, 2009; Lee *et al.*, 2018; Leung *et al.*, 2011; Litwin *et al.*, 2017; Verghese *et al.*, 2006; Wilson *et al.*, 2002). Most studies have adopted a composite measure of cognitive activities and less is known about the effects of a specific activity. To engage actively in daily life, activities may differ in their corresponding cognitive tasks and the amounts of intellectual stimulation required for active engagement (Ghisletta *et al.*, 2006). Besides, different activities might offset each other in their effects on cognitive function (Gallucci *et al.*, 2009; Lindstrom *et al.*, 2005; Lopes *et al.*, 2012; Lovden *et al.*, 2005). Research evidence for any specific activity is warranted.

Reading is a typical intellectual activity. Compared with other leisure activities, such as physical and social activities, it is more sedentary and isolated. Reading for leisure has proven to have health benefits for older people in prolonging life (Jacobs *et al.*, 2008), and cognition may mediate between reading and survival advantage (Bavishi *et al.*, 2016). Reading ability is strongly related to educational attainment in early life. It is unclear whether reading activity is protective against cognitive decline independent of education, and when reading ability could mediate between early education attainment and later-life cognitive function (Johnson *et al.*, 2006). There is little evidence that education could moderate the effect of reading on cognitive function (Lachman *et al.*, 2010). The aim of the study is twofold—first, to investigate the effect of reading on cognitive function in a representative Taiwanese older population who were followed up to 14 years. Second, we seek to investigate the differential effects of reading on preventing cognitive decline for older adults with different educational levels.

## Method

### Data

To explore the long-term relationship between cognitive activities and cognitive decline, we used data from the Taiwan Longitudinal Study on Aging (TLSA). The nation-wide longitudinal study started in 1989, and six sequential follow-ups were held in a 3- or 4-year interval between 1989 and 2011. In the first wave (1989), a representative sample of 4,049 older adults aged 60 and over was interviewed face-to-face with questionnaires with a 94% response rate. In the second wave (1993), 589 of the original participants were deceased, 301 were lost-to-follow-up, and five were incompletely interviewed, which resulted in 3,154 (78% of original respondents) completely interviewed. The second wave of surveys was the baseline for the study since cognitive functions were assessed for the first time. And, 2,946 out of 3,154 participants completed the assessment at baseline. In the 3rd (1996), 4th (1999), 5th (2003), and the 6th (2007) surveys, 2,669 (66%), 2,310 (59%), 1,743 (43%), and 1,268 (31%) of the first wave respondents completed these follow-ups. The third survey was not used in this study because its cognitive assessment was not comparable to those in other surveys. And, 1,973 participants had both a baseline survey and at least one follow-up survey with complete cognitive assessments. Participants who had severe cognitive impairment at baseline (*n* = 11) were excluded because the definition of cognitive decline in this study did not apply to them, and a total of 1,962 participants were included in the analysis (1,861 in 6-year, 1,330 in 10-year, and 818 in 14-year follow-up). Details of design and sampling for the surveys can be found elsewhere (Glei *et al.*, 2005; Lee *et al.*, 2012; Yen *et al.*, 2010). The data were officially released by and used abiding by the regulations of the Health and Welfare Data Science Center, Minister of Health and Welfare, Taiwan.

### Measurements

In the second wave of TLSA surveys, the Short Portable Mental Status Questionnaire (SPMSQ) was first used for assessing cognitive functions of participants. The instrument was also applied to the subsequent four waves. A SPMSQ test is composed of 10 items, which examine a respondent’s orientation to time and location, capacities of recalling personal information and current events, and a counting backward exercise. The SPMSQ was scored as the sum of errors, ranged from 0 to 10, which indicates the extent to which a respondent’s cognitive function is impaired. The Chinese version has been validated in the Taiwanese population (Hsiao *et al.*, 1994). However, all 10 items were adopted only in the second wave. From the 4th wave to 6th wave, nine items were used except asking a respondent’s birthday and only six items used in the 3rd wave. In the present study, we adopted nine items of SPMSQ from the 2nd, 4th, 5th, and 6th wave as the measurement of cognitive function. The 9-item SPMSQ scores (number of error responses) ranged from 0 to 9. Our preliminary analysis revealed that the 9-item SPMSQ might be less sensitive to the changes in cognitive functions for those who were developing cognitive impairments, although it has an acceptable and similar level of internal consistency as the 10-item version (Cronbach’s α of the 9-item and 10-item SPMSQ are 0.79 and 0.81 based on the data of 1993). Decline in cognitive function was defined as an increase of two or more SPMSQ scores between baseline (the 2nd wave) and subsequent surveys (Black and Rush, 2002; Fillenbaum *et al.*, 2001; Gray *et al.*, 2003; Hanlon *et al.*, 2004). We also conducted a sensitivity analysis that defined cognitive decline as an increase of one error or more in SPMSQ to understand if using a more sensitive criterion would affect the results of the analysis.

Reading was measured with a leisure-time activity scale in the 1993 survey, which asked respondents what leisure-time activities or entertainments they did in daily life when not working, and how often they did these activities. These activities included watching TV or listening to the radio, reading books/magazines/newspapers, worshiping, grand-parenting, playing chess/cards, visiting or hanging out with acquaintances, gardening, playing with pets, meditating, participating in group exercise, involvement in outdoor activities, or gambling. Weekly reading frequency was divided into four categories: “never,” “less than once a week,” “once or twice a week,” and “almost every day.” Due to the relatively smaller case numbers for “less than once a week” and “once or twice a week,” the former was merged with “never” as the “low reading group” and the latter merged with “almost every day” as the “high reading group.”

The baseline demographic and socioeconomic variables, including age, gender, ethnicity, education, marital status, and perceived financial status, were collected for multivariable analysis. Health behavior measures included smoking and alcohol drinking. The frequency of outdoor activities served as a surrogate for physical activity because there was no direct question indicating exercise in the 1993 survey. Activities of daily living (ADL) and instrumental activities of daily living (IADL) items consisted of a composite index to represent physical function with three levels: independent, IADL impairment, and ADL impairment. Self-reported diabetes mellitus (DM), stroke, and 10 other chronic diseases or health conditions were recorded. Twelve binary variables were created and coded as 1 to indicate the presence of DM, stroke, or health conditions and 0 for the absence of them. We left DM and stroke variables separately but summed the rest to an index indicating the number of comorbidities. Since reading is subject to vision, participants were categorized into with- and without-sight problems in terms of the presence of self-reported cataracts, glaucoma, retinal detachment, or unclear sight even when wearing glasses.

### Statistical analysis

Means and standard deviations were reported to describe the distributions of numeric variables, and frequencies and percentages to those of categorical variables. Contingency tables were established in the bivariate analysis and chi-square was used for testing statistical significance. In multivariate analysis, logistic regressions were performed to identify predictors of decline in cognitive function between baseline and follow-ups (i.e. 1999, 2003, and 2007, respectively.) Covariates for adjustments comprised age, sex, education, marital status, ethnicity, perceived financial status, smoking, alcohol drinking, outdoor activities, physical function, number of comorbidities, and sight problems. Diabetes and stroke and their preceding factors such as obesity, hypertension, hyperlipidemia were also the risk factors of cognitive decline (Baumgart *et al.*, 2015). The sequelae of diabetes and stroke such as retinopathy or acquired dyslexia may affect reading abilities. Therefore, diabetes and stroke were separated from other chronic conditions for controlling for potential confounding effects. Reading activities may represent abilities to engage in or be consequences of other intellectual or social leisure activities. We selected three other leisure activities, such as watching TV or listening to the radio, playing games (chess, cards, or mahjong), and visiting or hanging out with acquaintances for controlling for potential confounding effects between reading and cognitive outcomes. Comparing the high reading group to the low reading group in logistic regressions, a dummy variable was created and the low reading group served as reference. Higher frequency of reading is potentially a consequence of some precedent conditions like higher education, better baseline cognitive function, or sight. The high reading group might be over-represented in the sample, while the low reading group might be under-represented. Therefore, we further assessed the models with inverse probability weighting (IPW) to adjust for the potential selection bias (Hernan *et al.*, 2004). To understand the influence of the participants with cognitive impairments at baseline on the results, we conducted two parallel analyses: one for those with SPMSQ errors ≤2 at baseline and one for all eligible participants except SPMSQ errors ≥8 at baseline (because an increase of 2 errors did not apply to them using the 9-item SPMSQ). To investigate effects of reading on cognitive function decline with regard to education levels, two reading groups (high/low) and three education levels (illiterate and literate without formal education as “low,” primary school as “middle,” and junior high school and above as “high”) were re-categorized to form 2 × 3 = 6 groups. Interaction terms between education and reading were created to add in the logistic models where low reading groups and low education groups served as the references in comparison. Since one of the research questions is whether reading affects cognitive decline of older people with different education levels, we conducted planned comparisons without corrections between low and high reading groups at each education level using Wald tests. The significance level of all models was set at 0.05. All statistical analysis was performed using SAS 9.3 for Windows (SAS Institute, Cary, North Carolina).

This study was approved by the China Medical University & Hospital Research Ethics Committee, Taichung, Taiwan (CMUH103-REC3-057).

## Results

Demographics and other study variables are presented in Table 1. More than one-third (37%) of the sample indicated frequently reading at the baseline year. Those who frequently engaged in reading (more than twice a week) were men, junior high school or higher educational levels, mainlanders, living with spouse/partners, perceived sufficient in finances, nonsmokers, involved in more outdoor activities, without impaired physical functions, lower SPMSQ errors, without diabetes and sight problems, and lower numbers of chronic diseases.

**Table 1.** Characteristics of participants of the Taiwan Longitudinal Study on Aging, 1993–2007

	1993		1999		2003		2007	
variable	*n*	%	*n*	%	*n*	%	*n*	%
	2,946		1,861		1,330		818	
Mean age (SD)	71.0	(5.6)	69.8	(4.8)	68.8	(4.07)	68.1	(3.6)
Mean SPMSQ errors (SD)	1.20	(1.81)	1.08	(1.68)	1.23	(1.72)	1.36	(1.63)
Sex								
Male	1,679	57.0	1,028	55.2	723	54.4	445	54.4
Female	1,267	43.0	833	44.8	607	45.6	373	45.6
Education								
Illiterate	1,158	39.4	728	36.3	452	34.0	233	28.5
Literate without formal education	253	8.6	167	8.3	104	7.8	69	8.4
1–6 years	967	32.9	679	33.8	475	35.7	300	36.7
7–12 years	416	14.1	318	15.8	220	16.5	161	19.7
≥13 years	148	5.0	116	5.8	79	5.9	55	6.7
Reading frequency								
Never	1,762	59.8	1,059	56.2	716	53.8	398	48.7
Less than once a week	91	3.1	59	3.1	39	2.9	26	3.2
1–2 times a week	125	4.2	86	4.6	72	5.4	47	5.7
Almost every day	967	32.8	682	36.2	503	37.8	347	42.4
Ethnicity								
Holo	1,780	60.6	1,179	59.0	771	58.3	462	56.6
Hakka	456	15.5	307	15.4	195	14.8	125	15.3
Mainlander	655	22.3	488	24.4	341	25.8	225	27.6
Other	44	1.5	24	1.2	15	1.1	4	0.5
Marriage								
Living with spouse/partner	1,960	66.6	1,405	69.9	970	72.9	612	74.8
Separate	126	4.3	75	3.7	44	3.3	19	2.3
Without spouse	859	29.2	529	26.3	316	23.8	187	22.9
Perceived financial status								
Sufficient	1,007	34.2	645	34.5	475	35.7	312	38.1
Fair	1,571	53.4	1,030	55.1	721	54.2	441	53.9
Difficult	365	12.4	194	10.4	134	10.1	65	7.9
Smoking								
Non-smoker	2,068	70.2	1,353	71.7	968	72.8	619	75.7
Smoker	878	29.8	533	28.3	362	27.2	199	24.3
Drinking								
Non-alcohol drinker	2,407	81.7	1,515	80.3	1,050	78.9	631	77.1
Alcohol drinker	539	18.3	371	19.7	280	21.1	187	22.9
Outdoor Activities								
<2 per week	1,772	60.2	1,098	58.2	770	57.9	465	56.8
≥2 per week	1,173	39.8	788	41.8	560	42.1	353	43.2
Physical function								
Independent	1,519	52.1	1,106	59.9	843	63.8	557	68.7
IADL impairment	1,252	42.9	695	37.7	458	34.7	248	30.6
ADL impairment	147	5.0	45	2.4	20	1.5	6	0.7
Self-reported DM								
No	2,661	90.3	1,739	92.2	1,245	93.6	783	95.7
Yes	285	9.7	147	7.8	85	6.4	35	4.3
Self-reported stroke								
No	2,795	94.9	1,825	96.8	1,299	97.7	808	98.8
Yes	150	5.1	60	3.2	30	2.3	10	1.2
Sight								
No eye problem	1,793	60.9	1,183	62.7	857	64.4	548	67.0
Eye problem	1,153	39.1	703	37.3	473	35.6	270	33.0
Number of chronic diseases (SD)	1.40	(1.35)	1.35	(1.34)	1.36	(1.35)	1.28	(1.30)
Deceased from 1993 (%)	0	0.0	685	23.5	1259	42.7	1707	57.7
Follow-up years		8.91 (5.08)						

Results of logistic regressions on decline in cognitive function for those with SPMSQ errors ≤2 at baseline are displayed in Table 2. In the crude model, more reading, compared to less, was associated with a lower risk of cognitive function decline in the 6-year span, 10-year span, and 14-year span, respectively. Modeling with adjustment for age and sex increased the odds ratios (ORs) in 6-, 10- and 14-year spans to 0.40 (95% CI: 0.27–0.58), 0.46 (95% CI: 0.32–0.66) and 0.46 (95% CI: 0.31–0.67), respectively (Model 1). When education level was accounted for in Model 2, the ORs for those with a higher reading frequency were again elevated to 0.59 (95% CI: 0.38–0.92) in 6 years, 0.59 (95% CI: 0.39–0.91) in 10 years, and 0.60 (95% CI: 0.39–0.92) in 14 years. Model 3 with full adjustment presents the slightly reduced odds 0.54 (95% CI: 0.34–0.86), 0.58 (95% CI: 0.37–0.92) and 0.54 (95% CI: 0.34–0.86), respectively. In Model 4 with the illiterate excluded, the risks of cognitive decline for those with more reading were reduced to 0.50 (95% CI: 0.29–0.85) at 6 years, to 0.53 (95% CI: 0.32–0.88) at 10 years, and slightly increased to 0.55 (95% CI: 0.33–0.90) at 14 years. After controlling for three intellectual leisure activities in Model 5, the effect of reading was slightly weakened in 6 and 10 years, but slightly stronger in 14 years. In the model with IPW (Model 6), the ORs were changed mildly with reduced confidence intervals; they were 0.61 (95% CI: 0.42–0.88) in 6-year span, 0.54 (95% CI: 0.36–0.79) in 10-year span, and 0.51 (95% CI: 0.35–0.73) in 14-year span. The sensitivity analysis defining cognitive decline as an increase of one error or more revealed slightly attenuated effects of reading with full adjustments at 6 years (aOR: 0.69, 95% CI: 0.50–0.94), 10 years (aOR: 0.64, 95% CI: 0.46–0.90), and 14 years (aOR: 0.59, 95% CI: 0.39–0.88), compared with their counterparts in Model 3, probably caused by some substantial changes in cognitive function mixed up with an increase of one error occurred by chance (Supplementary Table S3).

**Table 2.** Effects of reading frequencies (higher versus lower) on decline in cognitive function among the TLSA participants with SPMSQ errors ≤ 2 at baseline

	6 years		10 years		14 years	
years of follow-up	aor	(95% ci)	aor	(95% ci)	aor	(95% ci)
Crude	0.27	(0.19–0.38)	0.35	(0.25–0.49)	0.43	(0.31–0.61)
Model 1	0.40	(0.27–0.58)	0.46	(0.32–0.66)	0.46	(0.31–0.67)
Model 2	0.59	(0.38–0.92)	0.59	(0.39–0.91)	0.60	(0.39–0.92)
Model 3	0.54	(0.34–0.86)	0.58	(0.37–0.92)	0.54	(0.34–0.86)
Model 4	0.50	(0.29–0.85)	0.53	(0.32–0.88)	0.55	(0.33–0.90)
Model 5	0.62	(0.39–1.00)	0.59	(0.38–0.93)	0.53	(0.33–0.84)
Model 6	0.61	(0.42–0.88)	0.54	(0.36–0.79)	0.51	(0.35–0.73)

Decline is defined by an increase of two or more SPMSQ errors between baseline and end-point years.

Model 1: Adjusted for age and sex.

Model 2: Model 1 plus educational level.

Model 3: Model 2 plus marital status, ethnicity, perceived financial status, smoking, alcohol drinking, outdoor activities, physical function, self-reported diabetes, stroke, number of comorbidities, sight problems, and the number of SPMSQ errors at baseline.

Model 4: Model 3 excluding the illiterate.

Model 5: Model 3 plus watching TV/listening to radio, playing games, and visiting or hanging out with acquaintances.

Model 6: Model 3 using inverse probability weighting method.

Including those with SPMSQ errors >2 at baseline in analysis, Table 3 shows the effect of reading on cognitive decline regardless of baseline cognitive function. In general, compared with the models corresponding to each other in Table 2, the effects of reading on cognitive decline were similar with slightly higher ORs. These results imply that the long-term effects of reading on cognitive decline in 10 years and 14 years with full adjustments were robust and mildly affected by baseline cognitive performance.

**Table 3.** Effects of reading frequencies on decline in cognitive function among the TLSA participants with inclusion of those with SPMSQ errors >2 at baseline

	6 years		10 years		14 years	
years of follow-up	aor	(95% ci)	aor	(95% ci)	aor	(95% ci)
Crude	0.28	(0.20–0.40)	0.40	(0.29–0.55)	0.52	(0.37–0.72)
Model 6	0.43	(0.30–0.63)	0.53	(0.37–0.76)	0.50	(0.34–0.73)
Model 7	0.62	(0.40–0.96)	0.68	(0.44–1.04)	0.64	(0.41–0.98)
Model 8	0.59	(0.37–0.93)	0.60	(0.38–0.93)	0.55	(0.35–0.86)
Model 9	0.49	(0.29–0.84)	0.53	(0.32–0.88)	0.57	(0.35–0.93)
Model 10	0.67	(0.42–1.07)	0.62	(0.39–0.98)	0.54	(0.34–0.81)
Model 11	0.63	(0.45–0.87)	0.55	(0.38–0.79)	0.56	(0.40–0.80)

Decline is defined by an increase of two or more SPMSQ errors between baseline and end-point years; participants having baseline SPMSQ errors ≥8 were not included since the definition of cognitive decline in terms of an increase of two or more errors did not apply to them.

Model 6: Adjusted for age and sex.

Model 7: Model 6 plus educational level.

Model 8: Model 7 plus marital status, ethnicity, perceived financial status, smoking, alcohol drinking, outdoor activities, physical function, self-reported diabetes, stroke, number of comorbidities, sight problems, and the number of SPMSQ errors at baseline.

Model 9: Model 8 excluding the illiterate.

Model 10: Model 8 plus watching TV/listening to radio, playing games, and visiting or hanging out with acquaintances.

Model 11: Model 8 using inverse probability weighting method.

**Figure 1.**
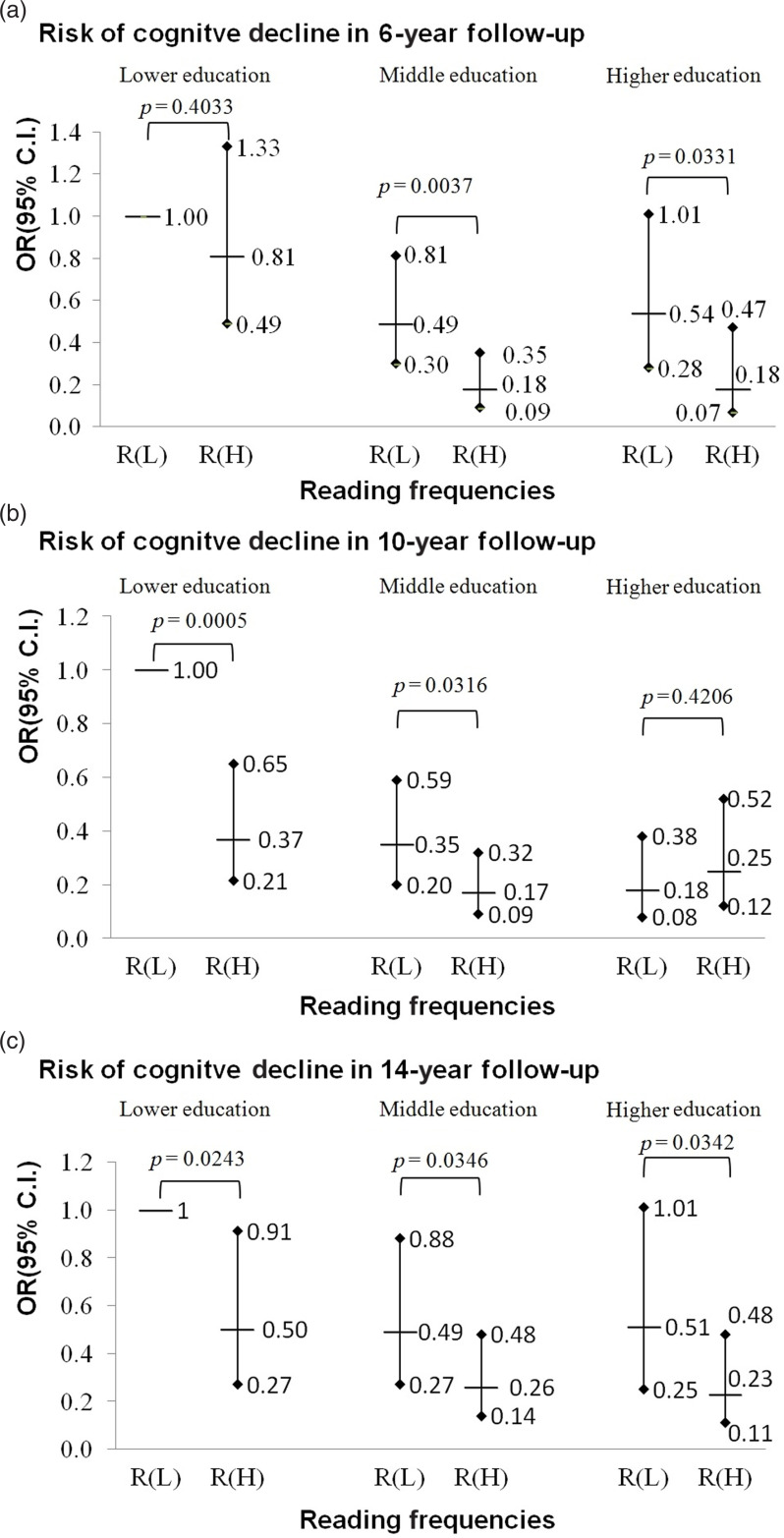
The combination effects of reading and education on the risk of cognitive decline at 6 years, 10 years, and 14 years of follow-ups in the TLSA participants. Results from logistic regression with interaction terms between education and reading based on Model 3 with adjustments for age, sex, ethnicity, marital status, financial status, smoking, alcohol drinking, outdoor activities, number of comorbidities, diabetes, stroke, sight, and the number of SPMSQ errors at baseline using the inverse probability weighting method. Participants with baseline SPMSQ ≤2 were included. Adjusted odds ratios and 95% confidence intervals are presented; *R*(*L*) denotes the groups with lower reading frequencies. *R*(*H*) denotes the groups with higher reading frequencies; Lower education refers to those who were not formally educated; middle education refers to 1–6 years of formal education; higher education refers to those who have had formal education for 7 years or more. Planned comparisons between low and high reading groups at the same education level are performed and *p* values of Wald tests for planned comparisons are reported.

The combined effects of reading and education on decline in cognitive function are presented in Figure 1. In general, those with a higher educational level were less likely to suffer decline in cognitive function regardless of the frequency of reading. In the 6-year follow-up, a person with more reading activities had an apparent lowered risk of cognitive decline among formally educated participants but did not among lower education participants. However, those with lower education could benefit most from reading activities in 10-year follow-up (aOR: 0.38, 95% CI: 0.21–0.66, *p* < 0.001), followed by those with middle education (high reading versus low reading, aOR: 0.17 versus 0.35; Wald test for comparison of aORs, *p* = 0.032), while the higher education group could benefit less (aOR: 0.12 versus 0.18; Wald test, *p* = 0.421). When participants were followed up 14 years after baseline, high frequencies versus lower frequencies of reading activities lead to significant decreases in odds of cognitive decline for those with lower education (aOR: 0.50 versus 1.00; Wald test, *p* = 0.024), middle education (0.26 versus 0.49; Wald test, *p* = 0.035), and high education (0.23 versus 0.51; Wald test, *p* = 0.034).

## Discussion

This study aims to appreciate the benefits of reading that deferred decline in cognitive function in later life in the Taiwanese older adults. Higher frequency of reading, i.e. twice or more a week, was associated with a reduced risk of decline in cognitive function over the long term—up to 14 years. With adjustment for relevant covariates and selection bias, our study also determines that frequent reading activities predict better cognitive outcomes at all educational levels, and the least formally educated might benefit most in the long term.

The finding that reading in daily life prevents cognitive decline for older people was in accordance with previous studies. Previous intervention studies assessing the effect of learning therapy, a training program consisting of reading aloud and arithmetic calculation which activates the dorsolateral prefrontal cortex, showed these mental activities could improve cognitive performance for patients with dementia in a randomized control trial (Kawashima *et al.*, 2005) and also for normal aged people in community settings (Uchida and Kawashima, 2008). However, the sample size was small and the follow-up time was short in their studies. A cross-sectional study on 668 Italian older adults aged over 70 years shows that regular reading was strongly associated with better cognitive performance when physical and social activities, but not educational level, were controlled for (Gallucci *et al.*, 2009). A US prospective study on 801 older clergypersons followed up to 7 years showed that a one-point increase in baseline cognitive activity score was associated with one-third less risk of Alzheimer’s disease and reduced decline in global cognitive function (Wilson *et al.*, 2002). The current study using samples from community dwellers in a general population repeatedly over a long time strengthens the evidence of reading benefit on reducing the risk of cognitive decline in later life.

Frequency of reading might be related to early education. The relationship between reading and reduced risk of cognitive decline might, therefore, reflect the education effect. Previous studies on early education achievement and cognitive function in later life were controversial. Some studies supported a positive relationship between education and cognitive performance of older adults (Black and Rush, 2002; Glei *et al.*, 2005; Wu *et al.*, 2011), while others did not (Christensen *et al.*, 2001; Muniz-Terrera *et al.*, 2009; Wilson *et al.*, 2002, 2019). These discrepancies between studies might result from different study populations, measurements, or modeling. Our study shows that, compared with illiterate participants, the risk of cognitive decline was significantly lower in older people who had attended school (Table 4); the higher the level of education, the lower the risk of cognitive decline. It echoes the findings of the study conducted by Liao *et al.* (2005) that the effect of education on cognitive reserve is cumulative. However, the gradient effect of education on cognitive decline gradually decreased with increasing time of follow-up. This may reflect the detrimental effect of age on the cognitive reserve produced by education in early life. The findings suggest that education can prevent cognitive decline in older people, and its effects may last for a long time.

**Table 4.** Effects of education on decline in cognitive function among the TLSA participants with SPMSQ errors ≤2 at baseline

	6 years ^†^		10 years ^†^		14 years ^†^	
years of follow-up	aor	(95% ci)	aor	(95% ci)	aor	(95% ci)
**Education years**						
Illiterate	Ref.		Ref.		Ref.	
Literate without formal education	0.72	(0.39–1.32)	0.57	(0.31–1.08)	0.89	(0.46–1.74)
1–6	0.43	(0.28–0.67)	0.41	(0.26–0.64)	0.50	(0.31–0.83)
7–12	0.35	(0.17–0.72)	0.43	(0.22–0.82)	0.45	(0.23–0.89)
≥13	0.17	(0.04–0.79)	0.32	(0.12–0.85)	0.34	(0.12–0.93)

† Adjusted for age, sex, marital status, ethnicity, reading frequencies, perceived financial status, smoking, alcohol drinking, outdoor activities, physical function, diabetes, stroke, number of comorbidities, sight and number of SPMSQ errors at baseline.

On the other hand, when models are adjusted for education, reading can significantly predict cognitive decline independent of education. In the IPW model, which amends selection bias due to a positive association between education and reading, those who read more had a reduced risk of cognitive decline with even greater statistical significance. The evidence from the current study supports that involvement in more reading is independently predictive of subsequent better cognitive function in later life.

Reading might benefit all education levels. When the follow-up time was shorter and the study participants were younger in the 6-year follow up, reading affected middle and higher education levels but not for the lower one. In the 10-year follow up, the effect of reading at the high education level disappeared, probably because some of them had developed preclinical dementia but showed no clinical symptoms at baseline and they still were able to read. In addition, higher levels of education may imply a later onset of some chronic diseases not captured at baseline. In the 14-year follow up, however, less educated participants were protected by reading as well as those with middle and higher education (Figure 1). Investment in education in childhood may help to increase cognitive reserve (Meng and D’Arcy, 2012; Stern, 2009), or cognitive capital as an economic metaphor (Rossor and Knapp, 2015), and prevent cognitive decline in later life. Like financial capital, cognitive capital can be depreciated (Richards and Deary, 2010). Engagement in reading might increase cognitive capital to resist aging-related cognitive function loss. Less-educated people may not have the intellectual skills of more-educated people for reading, and their prior cognitive ability before cognitive decline was lower; their capacity to benefit from reading activity was equivalent to, even higher than those of more-educated people in the long run. Our results echo a previous neuropathological study that found even very few years of education being able to improve cognitive reserve (Farfel *et al.*, 2013). This finding implies that, for policymakers, promoting reading for older adults as an intervention to improve successful aging might not introduce a new source to widen health inequity.

## Limitations

Missing data might challenge the representativeness of the sample and induce biased estimates. Most dropouts were due to death. Those who were more likely to drop out in follow-ups were those who were older, with more SPMSQ errors at baseline, less educated, less involved in reading, the Holo people, living without a spouse/partner, financially disadvantaged, smokers, non-alcohol drinkers, physically dependent, and having diabetes, stroke, or eye problems (data not shown). To answer whether the heterogeneity between missing and non-missing would bias the results, we employed the pattern-mixture models in which participants were regrouped in terms of their missing-data patterns (Hedeker and Gibbons, 2006; Little, 1995). In our study, dummy variables were created to indicate if observations were missing in 1999, 2003, or 2007. An auxiliary model with full adjustment plus these added terms was performed. It was found that only “missing in 2007” significantly impacts the effect of reading on 1993–2003 cognitive decline, which results in ORs in missing and non-missing subgroups, 0.92 (95% CI: 0.43–1.99) and 0.29 (95% CI: 0.15–0.58), respectively. This implies that reading was not protective of cognitive decline during 1993–2003 for those who would be deceased or dropped out before 2007. This might be because older persons with poor health or severe chronic conditions leading to death might also suffer from losses of physical and/or cognitive functions due to their morbidities. In this case, reading was probably restricted by their impaired functions; it was also unknown if reading could benefit those who have already developed cognitive impairment.

In this study, reading frequency was a self-reported measure. Respondents were asked to recall the frequency of reading activities, which may lead to recall bias, especially for older people who have already experienced cognitive impairment. There is no measurement of reading time in this study, so the relationship between reading time and cognitive function cannot be established. According to the Survey of Social Development: Time Use Survey, conducted by the Taiwanese government in 2004 (DGBAS, 2005), among those 65 years and older who have reading habits, newspaper readers spend 65 minutes a day, magazine readers 72 minutes a day, and casual book readers 99 minutes. We speculate that those with reading habits may read more than an hour a day.

We only measured reading at baseline. How long a participant has been practicing reading habits was not clear. According to the first survey of TLSA, 84% of participants with high reading frequencies in 1993 were also having high reading frequencies in 1989; 88% of participants with low reading frequencies in 1993 were having low reading frequencies in 1989 (see Supplementary Table S4); these figures show that reading habits were stable over long periods.

However, those who read more often at baseline might have read less in a follow-up. This might result in misclassification bias and underestimation of the effect of reading on cognitive function. Our qualitative conclusions would not be changed. On the other hand, those who read less at baseline might have more involvement due to participating in a post-school education program or reading groups—again reducing the differential in cognitive performance among subgroups with differential reading at baseline.

Weekly reading frequencies were categorized into two groups because of smaller sample sizes of “less than once a week” (R1) and “once or twice a week” (R2). We acknowledged that this combination was arbitrary and therefore performed sensitivity analysis. We combined R1 and R2 into “almost every day” (R3) and compared with “never” (R0), as well as combined R1 and R2 with R0 and compared with R3. The results of the former grouping show similar estimates to Model 3 in Table 2 (Supplementary Table S5). It implies that a minimum reading even less than once a week might also help. The latter grouping results in a reduced effect with a marginal significance in 6 years, partially a result from misclassification, although the effect remains significant in 10 and 14 years. From the results of the regrouping analysis, the grouping method has little effect on the estimates and does not affect the subsequent qualitative conclusions.

In conclusion, the current study presents the evidence that more engagement in reading independently predicted a reduced risk of cognitive decline in later life. Furthermore, the long-term favorable effect could be observed at all education levels; low education might not impede the beneficial effect of reading activities on cognitive function for older people. Promoting reading activity is a promising approach to improve cognitive health in community settings.

## Supplementary material

For supplementary material accompanying this paper visit https://doi.org/10.1017/S1041610220000812. click here to view supplementary material
