# Supplementary material for: Reading activity prevents long-term decline in cognitive function in older people: evidence from a 14-year longitudinal study
Source: Int Psychogeriatr. 2020 Jun 5;33(1):63–74. doi: 10.1017/S1041610220000812 (PMC8482376; doi:10.1017/S1041610220000812)
Supplement: Supplementary file 1 [file S1041610220000812sup001.docx]

Table S1. Cronbach’s α of the Short Portable Mental Status Questionnaire (SPMSQ) based on the Taiwan Longitudinal Study on Aging, 1993

| SPMSQ Item | N | Mean | SD | Correlation with total SPMSQ score | Cronbach’s α without the item |
| --- | --- | --- | --- | --- | --- |
| Home address | 2962 | 0.08 | 0.27 | 0.50 | 0.76 |
| Name of the place | 2956 | 0.02 | 0.13 | 0.29 | 0.79 |
| Date, month, & year | 2963 | 0.21 | 0.41 | 0.60 | 0.75 |
| Day of the week | 2964 | 0.09 | 0.28 | 0.50 | 0.76 |
| Mother's maiden name | 2961 | 0.09 | 0.29 | 0.32 | 0.78 |
| Name of current president | 2964 | 0.18 | 0.38 | 0.63 | 0.74 |
| Name of last president | 2963 | 0.26 | 0.44 | 0.61 | 0.74 |
| Age | 2963 | 0.04 | 0.19 | 0.29 | 0.79 |
| Birthday | 2964 | 0.20 | 0.40 | 0.50 | **0.79**^a^ |
| Backward count | 2962 | 0.25 | 0.44 | 0.51 | 0.76 |
|  |  |  |  |  |  |
| Cronbach’s α of all SPMSQ items^b^ |  |  |  |  |  |
| Raw | 0.81 |  |  |  |  |
| Standardized | 0.81 |  |  |  |  |

^a^Cronbach’s α for the 9-item SPMSQ without the birthday item

^b^Cronbach’s α for the 10-item SPMSQ

Table S2. The cross-classification of the 9-item and 10-item versions of Short Portable Mental Status Questionnaire (SPMSQ) for participants of the Taiwan Longitudinal Study on Aging, 1993

|  |  | **SPMSQ-10^a^** | | | | |
| --- | --- | --- | --- | --- | --- | --- |
|  |  | **0-2** | **3-4** | **5-7** | **8-10** | **Sum** |
| **SPMSQ-9^b^** |  |  |  |  |  |  |
| **0-2** | N | 2237 | 104 | 0 | 0 | 2341 |
|  | % | 100% | 26.2% | 0% | 0% | 79.5% |
| **3-4** | N | 0 | 293 | 95 | 0 | 388 |
|  | % | 0% | 73.8% | 35.2% | 0% | 13.2% |
| **5-7** | N | 0 | 0 | 175 | 22 | 197 |
|  | % | 0% | 0% | 64.8% | 52.4% | 6.7% |
| **8-9** | N | 0 | 0 | 0 | 20 | 20 |
|  | % | 0% | 0% | 0% | 47.6% | 0.7% |
| **Total** |  | 2237 | 397 | 270 | 42 | 2946 |

^a^The 10-item SPMSQ

^b^The 9-item SPMSQ

Table S3. Effects of reading frequencies on decline in cognitive function defined as an increase of one or more errors of the Short Portable Mental Status Questionnaire (SPMSQ) among participants of the Taiwan Longitudinal Study on Aging

| Years of Follow-up | 6 Years | | | | | | | |  | | 10 Years | | | | | | |  | | | 14 Years | | | | | |
| --- | --- | --- | --- | --- | --- | --- | --- | --- | --- | --- | --- | --- | --- | --- | --- | --- | --- | --- | --- | --- | --- | --- | --- | --- | --- | --- |
|  | AOR | ( | 95% C. I. | | | ) |  | | | AOR | | ( | 95% C. I. | | | ) |  | | | AOR | | ( | 95% C. I. | | | ) |
| Crude | 0.40 | ( | 0.31 | – | 0.50 | ) |  | 0.44 | | | | ( | 0.35 | – | 0.57 | ) |  | | 0.53 | | | ( | 0.40 | – | 0.71 | ) |
| Model S1 | 0.49 | ( | 0.38 | – | 0.63 | ) |  | 0.50 | | | | ( | 0.38 | – | 0.65 | ) |  | | 0.53 | | | ( | 0.38 | – | 0.74 | ) |
| Model S2 | 0.74 | ( | 0.55 | – | 1.00 | ) |  | 0.70 | | | | ( | 0.51 | – | 0.96 | ) |  | | 0.64 | | | ( | 0.44 | – | 0.94 | ) |
| Model S3 | 0.69 | ( | 0.50 | – | 0.94 | ) |  | 0.64 | | | | ( | 0.46 | – | 0.90 | ) |  | | 0.59 | | | ( | 0.39 | – | 0.88 | ) |
| Model S4 | 0.70 | ( | 0.50 | – | 0.99 | ) |  | 0.61 | | | | ( | 0.42 | – | 0.87 | ) |  | | 0.54 | | | ( | 0.34 | – | 0.83 | ) |
| Model S5 | 0.72 | ( | 0.53 | – | 0.99 | ) |  | 0.64 | | | | ( | 0.45 | – | 0.89 | ) |  | | 0.58 | | | ( | 0.39 | – | 0.88 | ) |
| Model S6 | 0.65 | ( | 0.50 | – | 0.85 | ) |  | 0.55 | | | | ( | 0.41 | – | 0.72 | ) |  | | 0.51 | | | ( | 0.37 | – | 0.72 | ) |

Decline is defined by an increase of one or more SPMSQ errors between baseline and end-point years

Model S1: Adjusted for age and sex

Model S2: Model S1 plus educational level

Model S3: Model S2 plus marital status, ethnicity, perceived financial status, smoking, alcohol drinking, outdoor activities, physical function, self-reported diabetes, stroke, number of comorbidities, sight and the number of SPMSQ errors at baseline

Model S4: Model S3 excluding the illiterate

Model S5: Model S3 plus watching TV/listening to radio, playing games and visiting or hanging out with acquaintances

Model S6: Model S3 using inverse probability weighting method

Table S4. Reading frequencies of participants of the Taiwan Longitudinal Study on Aging in 1989 and 1993

|  | Reading Frequency in 1989 | | | |  |
| --- | --- | --- | --- | --- | --- |
|  | ≤1 /week | | ≥2 /week | | Total |
| Reading Frequency in 1993 | N | % | N | % |  |
| ≤1 /week | 1774 | 87% | 254 | 13% | 2028 |
| ≥2 /week | 180 | 16% | 940 | 84% | 1120 |

Table S5. The effects of reading on cognitive decline based on different grouping of reading frequency

| Years of Follow-up | 6 Years | | | | | | | |  | | 10 Years | | | | | | |  | | | 14 Years | | | | | |
| --- | --- | --- | --- | --- | --- | --- | --- | --- | --- | --- | --- | --- | --- | --- | --- | --- | --- | --- | --- | --- | --- | --- | --- | --- | --- | --- |
|  | AOR | ( | 95% C. I. | | | ) |  | | | AOR | | ( | 95% C. I. | | | ) |  | | | AOR | | ( | 95% C. I. | | | ) |
| Reading Frequencies^✝^ |  |  |  |  |  |  |  |  | | | |  |  |  |  |  |  | |  | | |  |  |  |  |  |
| R1+R2+R3 vs. R0^a^ | 0.55 | ( | 0.35 | – | 0.88 | ) |  | 0.53 | | | | ( | 0.33 | – | 0.83 | ) |  | | 0.53 | | | ( | 0.33 | – | 0.84 | ) |
| R2+R3 vs. R0+R1^b^ | 0.54 | ( | 0.34 | – | 0.86 | ) |  | 0.58 | | | | ( | 0.37 | – | 0.92 | ) |  | | 0.54 | | | ( | 0.34 | – | 0.86 | ) |
| R3 vs. R0+R1+R2^c^ | 0.63 | ( | 0.39 | – | 1.03 | ) |  | 0.59 | | | | ( | 0.37 | – | 0.95 | ) |  | | 0.58 | | | ( | 0.37 | – | 0.92 | ) |

^✝^R0: never; R1: less than once a week; R2: 1-2 times a week; R3: almost daily;

^abc^Models adjusted for age, sex, education, marital status, ethnicity, perceived financial status, smoking, alcohol drinking, outdoor activities, physical function, self-reported diabetes, stroke, number of comorbidities, sight and the number of SPMSQ errors at baseline

Table S6. The Short Portable Mental Status Questionnaire (SPMSQ) scores of participants of the Taiwan Longitudinal Study on Aging, 1993. (N=2946)

| SPMSQ errors^✝^ | N | % |
| --- | --- | --- |
| 0-2 | 2341 | 79.5 |
| 3-4 | 388 | 13.2 |
| 5-7 | 197 | 6.7 |
| 8-9 | 20 | 0.7 |
